# Supplementary material for: Inter-Strain Epigenomic Profiling Reveals a Candidate IAP Master Copy in C3H Mice
Source: Viruses. 2020 Jul 21;12(7):783. doi: 10.3390/v12070783 (PMC7411935; doi:10.3390/v12070783)
Supplement: Supplementary file 1 [file viruses-12-00783-s001.zip › SupData1-revision01.docx]

Table of contents

[Supplementary figures 2](#_Toc44680939)

[Figure S1 2](#_Toc44680940)

[Figure S2 2](#_Toc44680941)

[Figure S3 3](#_Toc44680942)

[Figure S4 3](#_Toc44680943)

[Figure S5 4](#_Toc44680944)

[Figure S6 4](#_Toc44680945)

[Supplementary data 5](#_Toc44680946)

[C3H_HeJ IAP #60 full length copy - DNA 5](#_Toc44680947)

[C3H_HeJ full length IAP #60 copy - proteins 6](#_Toc44680948)

[DNA alignment of the full-length copy between C3H/HeJ (IAP #60) and the 129 genome 7](#_Toc44680949)

# Supplementary figures

## Figure S1


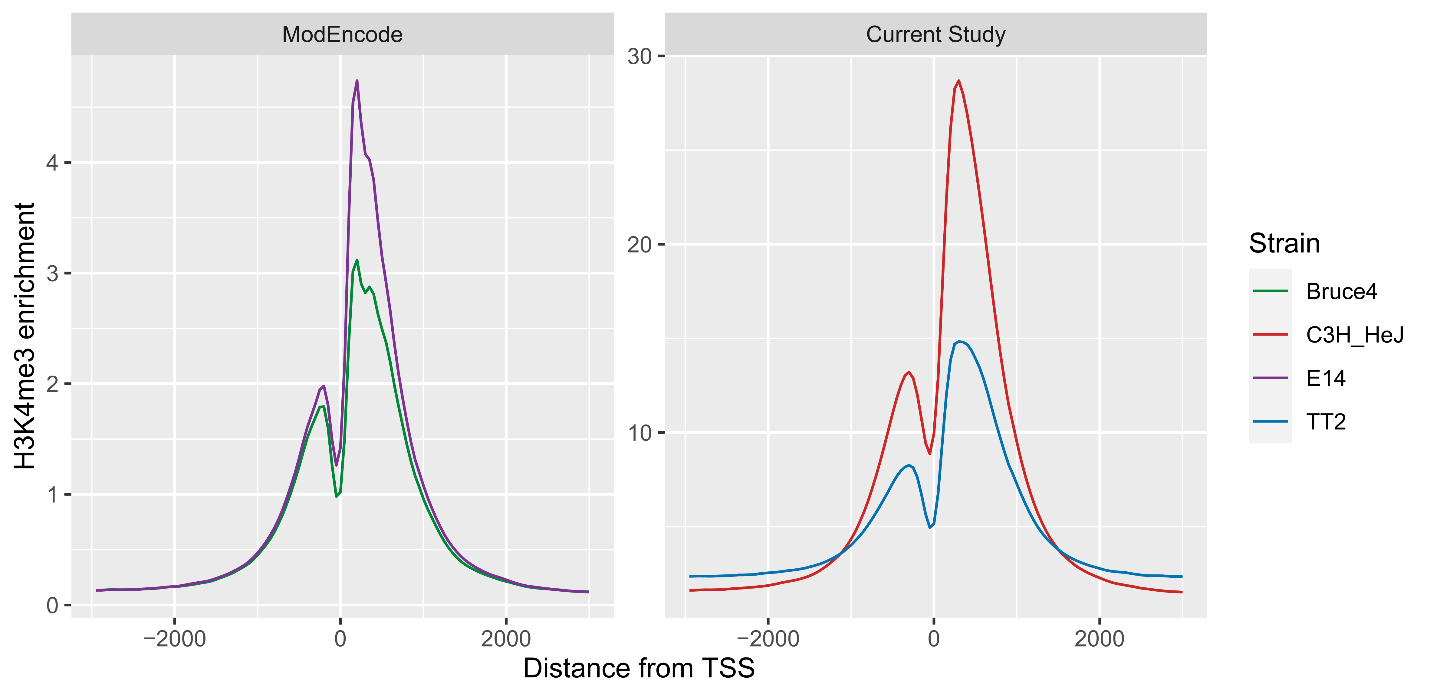


Figure S1. H3K4me3 enrichment around TSS for the datasets produced in this manuscript ( C3H/HeJ and TT2) and for the ModEncode datasets (Bruce4 and E14).

## Figure S2


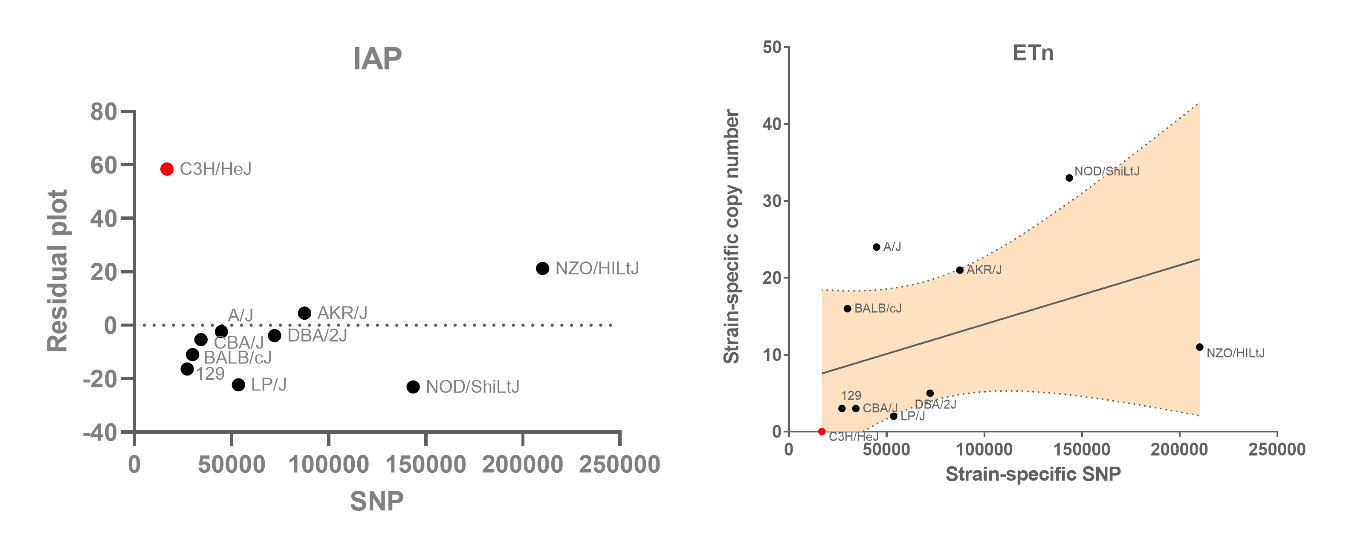


Figure S2. Supporting data for Figure 1. Left panel shows residuals standard deviation for private IAP copies versus private SNPs (see linear regression, Figure 1C). Right panel depicts private ETn copy number versus private SNPs across mouse strains.

## Figure S3


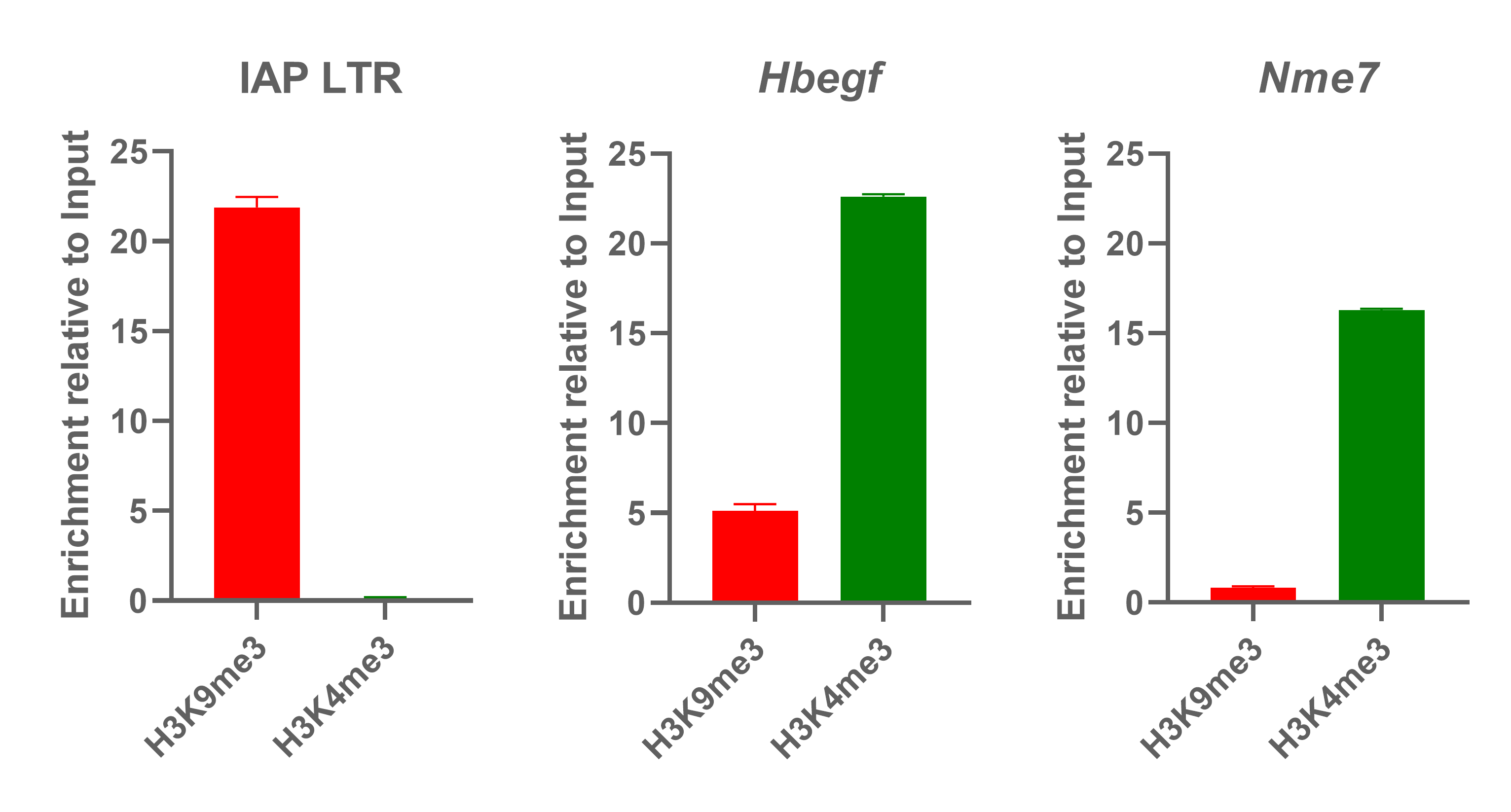


Figure S3: ChIP-qPCR of H3K9me3 and H3K4me3 for all IAP copies (non-specific copy primers targeting LTR-int), and for two H3K4me3 copies found only in C3H/HeJ, located inside the *Hbegf* (IAP # 30) and *Nme7* (IAP #3) genes (see Table S2).

## Figure S4


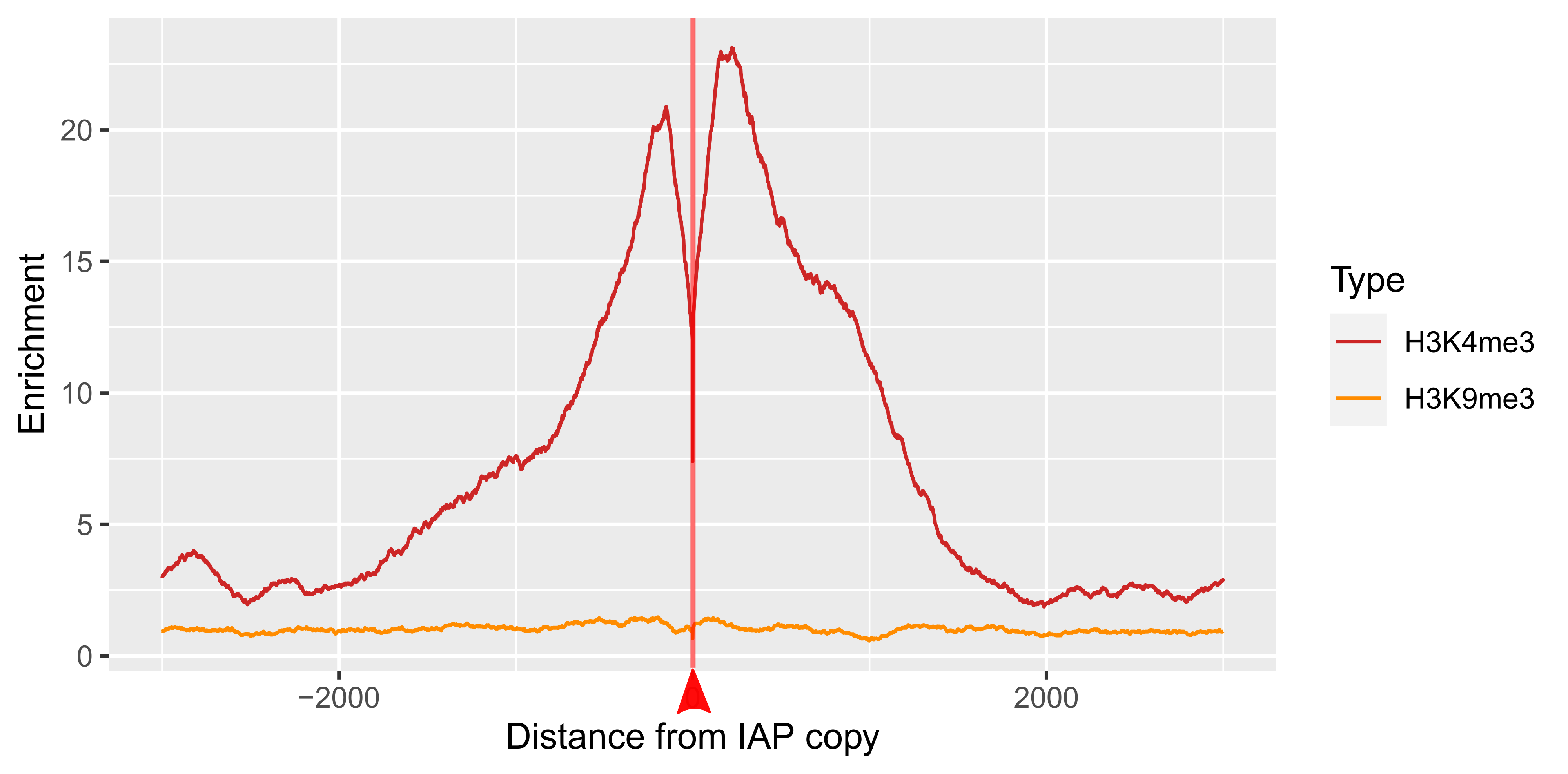


Figure S4. H3K4me3 and H3K9me3 enrichment in flanking sites of all H3K4me3-marked C3H/HeJ copies present in Table S2. Figure shows lack of H3K9me3 in in K4me3 flanking sites.

## Figure S5


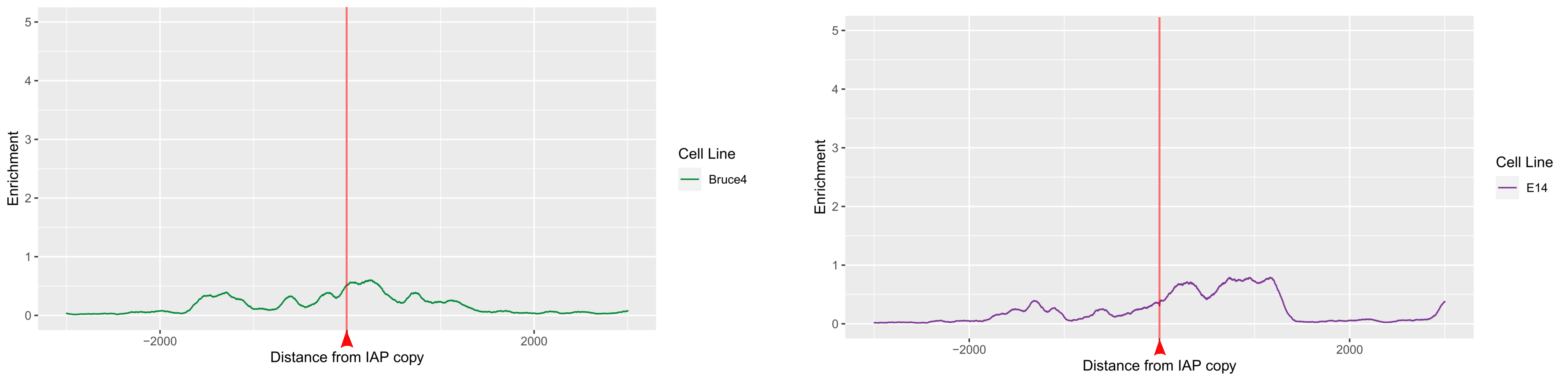


Figure S5. H3K4me3 enrichment in empty sites of Bruce4 and E14. Figure show lack of H3K4me3 in empty sites.

## Figure S6


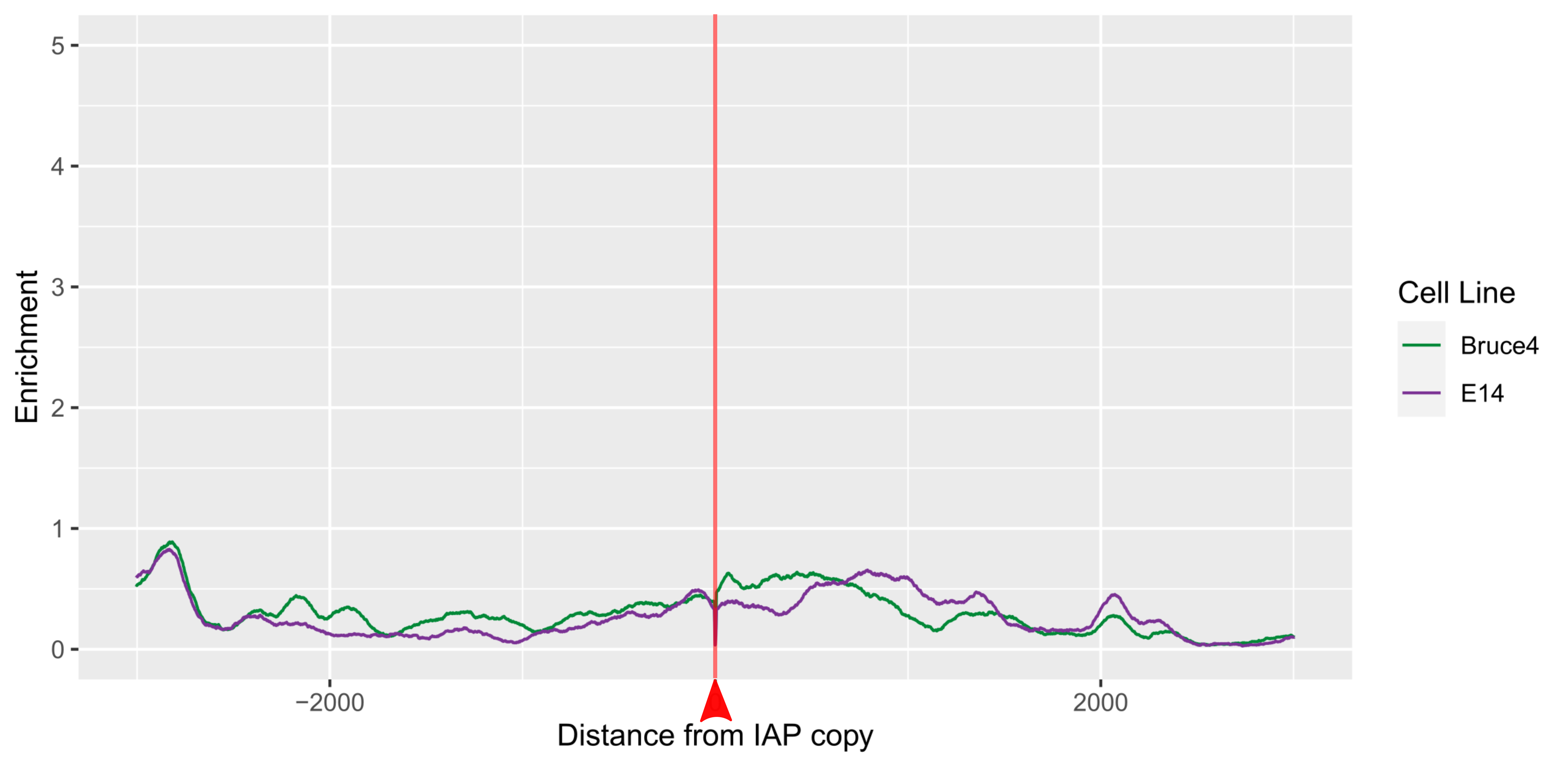


Figure S6. H3K4me3 enrichment in flanking sites of IAP copies present in C3H/HeJ, C57BL/6 and 129 of Bruce4 and E14. Figure show lack of H3K4me3 in flanking sites of common copies.

# Supplementary data

## C3H_HeJ IAP #60 full length copy - DNA

Legend

LTR PBS GAG SHIN PRO POL

>C3H-FL

TGTGGGAAGCCGCCCCCACATTCGCCGTCACAAGATGGCGCTGACATCCTGTGTTCTAAGTTGGTAAACAAATAATCTGCGCATGAGCCAAGGGTATTTACGACTACTTGTACTCTGTTTTTCCCGTGAACGTCAGCTCGGCCATGGGCTGCAGCCAATCAGGGAGTGATGCGCCCTAGGCAATGGTTGTTCTCTTTAAAATAGAAGGGGTTTCGTTTTTCTCGCTCTCTTGCTTCCCTCTCTTGCTTCTTACACTCTGGCCCGATAAAGATATAAGCAATAAAGCTTTGCCGTAGAAGATTCTGGTTGTTGTGTTCTTCCTGGCCGGTCGTGAGAACGCGTCGAATAACAATTGGTGCCGAATTCCGGGACGAGAAAATCCGGGACGAGAAAAAACTCCGGACTGGCGCAGGAGGGATACTTCATTTCAGAACCAGAACTACGGATCACGTTTATAAAGGTTCCCGTAACACAGACTGTTGAGAAGGATCAACTGCCGAATTCAGACTCATCAGCTGGGGAACGACGGTGATAAAGGTCCCGTAAGCAGACTGTTAAGAAGGATTCAACTGTATGAATTCAGAACTTTTCAGCTGGGGAACGAGAGTACCAGTGAGTATGTTTGGCCTTGAATTTTTTCTGGTGTTAGGAGCCCTTTTGTTCCTTTTCACATGTTATATAGTGGTTAAGGCAGGGCTGAAAATTCTGGATGAAATTCAGGGCAGTCTATCAGAAGTAAAGCGGGGAGAGAGAGTAGGAGCAAGGAGAAACGGTAAGTATACAGGCCTTTCCAAGGGTCTTGAACCCGAGGAAAAGTTAAGGTTAGGTAGGAATACCTGGAGAGAGATTAGAAGAAAAAGAGGAAAAAGGGAAAAGAAAAAAGATCGATTAGCGGAGGTCTCTAGGAGATACTCGTCACTAGATGAGCTCAGGAAGCCAGCTCTTAGTAGCTCTGAAGCAAGTGAAGAATCCTCCTCTGAGGAAACAGACTGGGAGGAAGAAGCAGCCCATTACCAGCCAGCTAATTGGTCAAGAAAAAAGCCAAAAGCGGCTGGCGAAAGTCAGCGTACTGTTCAACCTCCCGGCAGTCGGTTTCAAGGTCCGCCCTATGCGGAGCCCCCGCCCTGCGTAGTGCGTCAGCAATGCGCAGAGAGGCAATGCGCAGAGAGGTGCGCAGAGAGGCAGTGCGCAGACAGGTGCGCAGAGAGGCAGTGCGCAGAGAGGCAGTGCGCAGACTCATTCATTCCCCGAGAGGAACAAAAGAAAATAGAACAGGCATTTCCAGTCTTTGAAGGAGCCGAGGGTGGGCGTGTCCACGCTCCGGTAGAATACGTACAGATTAAGGAAATTGCCGAGTCGGTTCGTAAATACGGAACCAATGCTAATTTCACCTTGGTGCAGTTAGACAGGCTCGCTGGTATGGCACTAACGCCTGCTGATTGGCAGACGGTTGTAAAAGCCGCTCTTCCTAGTATGGGCAAATATATGGAATGGAAAGCGCTTTGGCACGAAGCTGCACAGGCGCAGGCCCGAGCAAACGCAGCTGCTTTGACTCCAGAGCAGATAGATTGGACTTTTGACTTGTTAACGGGTCAGGGGGCTTATTCTGCTGATCAGACAAGCTACCATTGGGGAGCTTATGCCCAGATTTCTTCCACGGCTATTAGGGCCTGGAAGGCGCTCTCTCGAGCAGGTGAAACCACTGGTCAGTTAACAAAAATAATCCAGGGACCTCAGGAATCTTTCTCAGATTTTGTGGCCAGAATGACAGAGGCAGCAGAGCGTATTTTTGGAGAGTCAGAGCAAGCTGCGCCTCTCATAGAACAGCTAATCTACGAGCAAGCCACAAAGGAGTGCCGAGCGGCCATAGCCCCAAGAAAGAACAAAGGCTTACAAGACTGGCTCAGGGTCTGTCGAGAGCTTGGGGGACCCCTTAGCAATGCAGGTTTAGCGGCTGCCATCCTTCAATCCCAAAACCGCTCCATGGGCAGAAATGATCAGAGGACATGTTTTAACTGCGGAAAGCCTGGGCATTTTAAGAAAGATTGCAGAGCTCCAGATAAACAGGGAGGGGCTCTCACTCTTTGCTCTAAGTGTGGCAAGGGTTATCATAGAGCTGACCAGTGTCGCTCTGTGAGGGATATAAAGGGCAGAATTCTTCCCCCACCTGATAGTCAATCAGCTGATGTGCCAAAAAACGGGTCACCGGGCCCTCGGTCCCAGGGCCCTCAAAGATATGGGAACCGGTTTGTCAGGACCCAGGAAGCAGTCAGAGAGACGACCCAGGAAGACCCACAAGGGTGGACCTGCGTGCCGCCTCCGACTTCCTATTAATGCCTCAAATGAGTATTCAGCCGGTGCCGGTGGAGCCTATACCATCCTTGCCCCCGGGAACCATGGGCCTTATTCTCGGCCGAGGTTCACTCACCTTGCAGGGCTTAGTAGTCCACCCTGGAATTATGGATTGTCAACATTCCCCTGAAATACAGGTCCTGTGCTCAAGCCCTAAAGGCGTTTTTTCTATTAGTAAAGGAGATAGGATAGCTCAGCTGCTGCTCCTCCCTGATAATACCAGGGAGAAATCTGCAGGACCTGAGATAAAGAAAATGGGCTCCTCAGGAAATGATTCTGCCTATTTGGTTGTATCTTTAAATGATAGACCTAAGCTCCGCCTTAAGATTAATGGAAAAGAGTTTGAAGGCATCCTTGATACCGGAGCAGATAAAAGTATAATTTCTACACATTGGTGGCCCAAAGCATGGCCCACCACAGAGTCATCTCATTCATTACAGGGCCTAGGATATCAATCATGTCCCACTATAAGCTCCGTTGCCTTGACGTGGGAATCCTCTGAAGGGCAGCAAGGGAAATTCATACCTTATGTGCTCCCACTCCCGGTTAACCTCTGGGGAAGGGATATTATGCAGCATTTGGGCCTTATTTTGTCCAATGAAAACGCCCCATCAGGAGGGTATTCAGCTAAAGCAAAAAATATCATGGCAAAGATGGGTTATAAAGAAGGAAAAGGGTTAGGACATCAAGAACAGGGAAGGATAGAGCCCATCTCACCTAATGGAAACCAAGACAGACAGGGTCTGGGTTTTCCATAGCGGCCATTGGGGCAGCACGACCCATACCATGGAAAACAGGGGACCCAGTGTGGGTTCCTCAATGGCACCCATCCTCTGAAAAACTAGAAGCTGTGATTCAACTGGTAGAGGAACAATTAAAATTAGGCCATATTGAACCCTCTACCTCACCTTGGAATACTCCAATTTTTGTAATTAAGAAAAAGTCAGGAAAGTGGAGACTGCTCCATGACCTCAGAGCCATTAATGAGCAAATGAACTTATTTGGCCCAGTACAGAGGGGTCTCCCTGTACTTTCCGCCTTACCACGTGGCTGGAATTTAATCATTATAGATATTAAAGATTGTTTCTTTTCTATACCTTTGTGTCCAAGGGATAGGCCCAGATTTGCCTTTACCATCCCCTCTATTAATCACATGGAACCTGATAAGAGGTATCAATGGAAGGTCTTACCACAGGGAATGTCCAATAGTCCTACTATGTGTCAACTTTATGTACAAGAAGCTCTTTTGCCAGTGAGGGAACAATTCCCCTCTTTAATTTTGCTCCTTTACATGGATGACATCCTCCTGTGCCATAAAGACCTTACCATGCTACAAAAGGCATATCCTTTTCTACTTAAAACTTTAAGTCAGTGGGGTCTACAGATAGCCACAGAAAAGGTCCAAATTTCTGATACAGGACAATTCTTGGGCTCTGTGGTGTCCCCAGATAAGATTGTGCCCCAAAAGGTAGAGATAAGAAGAGATCACCTCCATACCTTAAATGATTTTCAAAAGCTGTTGGGAGATATTAATTGGCTCAGACCCTTTTTAAAGATTCCTTCTGCTGAATTAAGGCCTTTGTTTAGTATTTTAGAAGGAGATCCTCATATCTCCTCCCCTAGGACTCTTACTCTAGCTGCTAACCAGGCCTTACAAAAAGTGGAAAAAGCCTTACAGAATGCACAATTACAACGTATTGAGGATTCGCAGCCTTTCAGTTTGTGTGTCTTTAAGACAGCACAATTGCCAACTGCAGTTTTGTGGCAAAATGGGCCATTGTTGTGGATCCATCCAAACGTATCCCCAGCTAAAATAATAGATTGGTATCCTGATGCAATTGCACAGCTTGCCCTTAAAGGCCTAAAAGCAGCAATCACCCACTTTGGGCAAAGTCCATATCTTTTAATTGTACCTTATACCGCTGCACAGGTTCAAACCTTGGCAGCCGCATCTAATGATTGGGCAGTTTTAGTTACCTCCTTTTCAGGAAAAGTAGATAACCATTATCCAAAGCATCCAATCTTACAGTTTGCCCAAAATCAATCTGTTGTGTTTCCACAAATAACAGTAAGAAACCCACTTAAAAATGGGATTGTGGTATATACTGATGGATCAAAAACTGGCATAGGTGCCTATGTGGCTAATGGTAAAGTGGTATCCAAACAATATAATGAAAATTCACCTCAAGTGGTAGAATGTTTAGTGGTCTTAGAAGTTTTAAAAACCTTTTTAGAACCCCTTAATATTGTGTCAGATTCCTGTTATGTGGTAAATGCAGTAAATCTTTTAGAAGTGGCCGGAGTGATTAAGCCTTCCAGTAGAGTTGCCAATATTTTTCAGCAGATACAATTAGTTTTGTTATCTAGAAGATCTCCTGTTTATATTACTCATGTTAGAGCCCATTCAGGCCTACCTGGCCCCATGGCTCTGGGAAATGATTTGGCAGATAAGGCCACTAAAGTGGTGGCTGCTGCCCTATCATCCCCGGTAGAGGCTGCAAGAAATTTTCATAATAATTTTCATGTGACGGCTGAAACATTACGCAGTCGTTTCTCCTTGACAAGAAAAGAAGCCCGTGACATTGTTACTCAATGTCAAAGCTGCTGTGAGTTCTTGCCAGTTCCTCATGTGGGAATTAACCCACGCGGTATTCGACCTCTACAGGTCTGGCAAATGGATGTTACACATGTTTCTTCCTTTGGAAAACTTCAATATCTCCATGTGTCCATTGACACATGTTCTGGCATCATGTTTGCTTCTCCGTTAACTGGAGAAAAAGCCTCACATGTGATTCAACATTGTCTTGAGGCATGGAGTGCTTGGGGGAAACCCAGACTCCTTAAGACTGATAATGGACCAGCTTATACGTCCCAAAAATTTCAGCAGTTCTGCCGTCAGATGGACGTAACCCACCTGACTGGACTTCCATACAACCCTCAAGGACAGGGTATTGTTGAGCGTGCGCATCGCACCCTCAAAGCCTATCTTATAAAACAGAAGAGGGGAACTTTTGAGGAGACTGTACCCCGAGCACCAAGAGTGTCGGTGTCTTTGGCACTCTTTACACTCAATTTTTTAAATATTGATGCTCATGGCCATACTGCGGCTGAACGTCATTGTTCAGAGCCAGATAGGCCCAATGAGATGGTTAAATGGAAAAATGTCCTTGATAATAAATGGTATGGCCCGGATCCTATCTTGATAAGATCCAGGGGAGCTATCTGTGTTTTCCCACAGAATGAAGACAACCCATTTTGGGTACCAGAAAGACTCACCCGAAAAATCCAGACTGACCAAGGGAATACTAATGTCCCTCGTCTTGGTGATGTCCAGGGCGTCAATAATAAAGAGAGAGCAGCGTTGGGGGATAATGTCGACATTTCCACTCCCAATGACGGTGATGTATAATGCTCAAGTATTCTCCTGCTTTTTTACCACTAACTGGGAACTGGGTTTGGCCTTAATTCAGACAGCCTTGGTTCTGTCTGGACAGGTCCAGATGACTGACACCATTAACACTTTGTCAGCCTCAGTGACTACAGTCATAGATAAACAGGCCTCAGCTAATGTCAAGATACAGAGAGGTCTCATGCTGGTTAATCAACTCATAGATCTTGTCCAGATACAACTAGATGTATTATGACAAATAACTCAGCAGGGATGTGAACAAAAGTTTCCGGGATTGTGTGTTATTTCCATTCAGTATGTTAAATTTACTAGGGCAGCTAATTTGTCAAAAAGTCTTTTTCAGTATATGTTACAGATCCTTCGAGAATTGAGACTTCAGGTCAACTCCACGCGCTTGGACCTGTCGCTGACCAAAGGATTACCCAATTGGATCTCCTCAGCATTTTCTTTCTTTAAAAAATTGGGTGGGATTAATATTATTTGGAGATACACGTTGCTGTGGATTAGTGTTGCTTCTTTGATTGGTCTGTAAGCTTAAGGCCCAAACTAAGAGAGACAAGGTGGTTATTGCCCAGGCGCTTGCAGGACTAGAACATGGAGCTTCCCCTGATATATCTATGCTTAAGCAATAGGTCGCTGGCCACTCAGCTCTTATATCTCACGAGGCTAGTCTCATTGCACGAGGTAGAGTGAGTGTGCTTCAGCAGCCCGAGAGAGTTGCAAGGCTAAGCACTGCAGTAGAAGGGCTCTGCGGCACATATGAGCCTATTCTAGGGAGACATGTCATCTTTCATGAAGGTTCAGTGTCCTAGTTCCCTTCCCCCAGGCAAAACGACACGGGAGCAGGTCAGGGTTGCTCTGGGTAAAAGCCTGTAAGCCTAAGAGCTAATCCTGTACATGGCTCCTTTACCTACACACTGGGGATTTGACCTCTATCTCCACTCTCATTAATATGGGTGGCCTATTTGCTCTTATTAAAAGAAAAAGGGGGAACTGTGGGAAGCCGCCCCCACATTCGCCGACACAAGATGGCGCTGACATCCTGTGTTCTAAGTTGGTAAACAAATAATCTGCGCATGAGCCAAGGGTATTTACGACTACTTGTACTCTGTTTTTCCCGTGAACGTCAGCTCGGCCATGGGCTGCAGCCAATCAGGGAGTGATGCGCCCTAGGCAATGGTTGTTCTCTTTAAAATAGAAGGGGTTTCGTTTTTCTCGCTCTCTTGCTTCCCTCTCTTGCTTCTTACACTCTGGCCCGATAAAGATATAAGCAATAAAGCTTTGCCGTAGAAGATTCTGGTTGTTGTGTTCTTCCTGGCCGGTCGTGAGAACGCGTCGAATAACA

## C3H_HeJ full length IAP #60 copy - proteins

>GAG

MNSELFSWGTRVPVSMFGLEFFLVLGALLFLFTCYIVVKAGLKILDEIQGSLSEVKRGERVGARRNGKYTGLSKGLEPEEKLRLGRNTWREIRRKRGKREKKKDRLAEVSRRYSSLDELRKPALSSSEASEESSSEETDWEEEAAHYQPANWSRKKPKAAGESQRTVQPPGSRFQGPPYAEPPPCVVRQQCAERQCAERCAERQCADRCAERQCAERQCADSFIPREEQKKIEQAFPVFEGAEGGRVHAPVEYVQIKEIAESVRKYGTNANFTLVQLDRLAGMALTPADWQTVVKAALPSMGKYMEWKALWHEAAQAQARANAAALTPEQIDWTFDLLTGQGAYSADQTSYHWGAYAQISSTAIRAWKALSRAGETTGQLTKIIQGPQESFSDFVARMTEAAERIFGESEQAAPLIEQLIYEQATKECRAAIAPRKNKGLQDWLRVCRELGGPLSNAGLAAAILQSQNRSMGRNDQRTCFNCGKPGHFKKDCRAPDKQGGALTLCSKCGKGYHRADQCRSVRDIKGRILPPPDSQSADVPKNGSPGPRSQGPQRYGNRFVRTQEAVRETTQEDPQGWTCVPPPTSY

>PRO

MPQMSIQPVPVEPIPSLPPGTMGLILGRGSLTLQGLVVHPGIMDCQHSPEIQVLCSSPKGVFSISKGDRIAQLLLLPDNTREKSAGPEIKKMGSSGNDSAYLVVSLNDRPKLRLKINGKEFEGILDTGADKSIISTHWWPKAWPTTESSHSLQGLGYQSCPTISSVALTWESSEGQQGKFIPYVLPLPVNLWGRDIMQHLGLILSNENAPSGGYSAKAKNIMAKMGYKEGKGLGHQEQGRIEPISPNGNQDRQGLGFP

>POL

MNLFGPVQRGLPVLSALPRGWNLIIIDIKDCFFSIPLCPRDRPRFAFTIPSINHMEPDKRYQWKVLPQGMSNSPTMCQLYVQEALLPVREQFPSLILLLYMDDILLCHKDLTMLQKAYPFLLKTLSQWGLQIATEKVQISDTGQFLGSVVSPDKIVPQKVEIRRDHLHTLNDFQKLLGDINWLRPFLKIPSAELRPLFSILEGDPHISSPRTLTLAANQALQKVEKALQNAQLQRIEDSQPFSLCVFKTAQLPTAVLWQNGPLLWIHPNVSPAKIIDWYPDAIAQLALKGLKAAITHFGQSPYLLIVPYTAAQVQTLAAASNDWAVLVTSFSGKVDNHYPKHPILQFAQNQSVVFPQITVRNPLKNGIVVYTDGSKTGIGAYVANGKVVSKQYNENSPQVVECLVVLEVLKTFLEPLNIVSDSCYVVNAVNLLEVAGVIKPSSRVANIFQQIQLVLLSRRSPVYITHVRAHSGLPGPMALGNDLADKATKVVAAALSSPVEAARNFHNNFHVTAETLRSRFSLTRKEARDIVTQCQSCCEFLPVPHVGINPRGIRPLQVWQMDVTHVSSFGKLQYLHVSIDTCSGIMFASPLTGEKASHVIQHCLEAWSAWGKPRLLKTDNGPAYTSQKFQQFCRQMDVTHLTGLPYNPQGQGIVERAHRTLKAYLIKQKRGTFEETVPRAPRVSVSLALFTLNFLNIDAHGHTAAERHCSEPDRPNEMVKWKNVLDNKWYGPDPILIRSRGAICVFPQNEDNPFWVPERLTRKIQTDQGNTNVPRLGDVQGVNNKERAALGDNVDISTPNDGDV

## DNA alignment of the full-length copy between C3H/HeJ (IAP #60) and the 129 genome

In bold underlined, the SHIN region

**C3H** 1 TGTGGGAAGCCGCCCCCACATTCGCCGTCACAAGATGGCGCTGACATCCTGTGTTCTAAGTTGGTAAACAAATAATCTGCGCATGAGCCAAGGGTATTTA

**129** 1 TGTGGGAAGCCGCCCCCACATTCGCCGTCACAAGATGGCGCTGACATCCTGTGTTCTAAGTTGGTAAACAAATAATCTGCGCATGAGCCAAGGGTATTTA

**C3H** 101 CGACTACTTGTACTCTGTTTTTCCCGTGAACGTCAGCTCGGCCATGGGCTGCAGCCAATCAGGGAGTGATGCGCCCTAGGCAATGGTTGTTCTCTTTAAA

**129** 101 CGACTACTTGTACTCTGTTTTTCCCGTGAACGTCAGCTCGGCCATGGGCTGCAGCCAATCAGGGAGTGATGCGCCCTAGGCAATGGTTGTTCTCTTTAAA

**C3H** 201 ATAGAAGGGGTTTCGTTTTTCTCGCTCTCTTGCTTCCCTCTCTTGCTTCTTACACTCTGGCCCGATAAAGATATAAGCAATAAAGCTTTGCCGTAGAAGA

**129** 201 ATAGAAGGGGTTTCGTTTTTCTCGCTCTCTTGCTTCCCTCTCTTGCTTCTTACACTCTGGCCCGATAAAGATATAAGCAATAAAGCTTTGCCGTAGAAGA

**C3H** 301 TTCTGGTTGTTGTGTTCTTCCTGGCCGGTCGTGAGAACGCGTCGAATAACAATTGGTGCCGAATTCCGGGACGAGAAAATCCGGGACGAGAAAAAACTCC

**129** 301 TTCTGGTTGTTGTGTTCTTCCTGGCCGGTCGTGAGAACGCGTCGAATAACAATTGGTGCCGAATTCCGGGACGAGAAAATCCGGGACGAGAAAAAACTCC

**C3H** 401 GGACTGGCGCAGGAGGGATACTTCATTTCAGAACCAGAACTACGGATCACGTTTATAAAGGTTCCCGTAACACAGACTGTTGAGAAGGAT-CAACTGCCG

**129** 401 GGACTGGCGCAGGAGGGATACTTCATTTCAGAACCAGAACTACGGATCACGTTTATAAAGGTTCCCGTAACACAGACTGTTGAGAAGGATTCAACTGCCG

**C3H** 500 AATTCAGA-CTCATCAGCTGGGGAACGACGGTGATAAAGGT-CCCGTAA-GCAGACTGTTAAGAAGGATTCAACTGTATGAATTCAGAACTTTTCAGCTG

**129** 501 AATTCAGAACTCATCAGCTGGGGAACGACGGTGATAAAGGTTCCCGTAAAGCAGACTGTTAAGAAGGATTCAACTGTATGAATTCAGAACTTTTCAGCTG

**C3H** 597 GGGAACGAGAGTACCAGTGAGTATGTTTGGCCTTGAATTTTTTCTGGTGTTAGGAGCCCTTTTGTTCCTTTTCACATGTTATATAGTGGTTAAGGCAGGG

**129** 601 GGGAACGAGAGTACCAGTGAGTATGTTTGGCCTTGAATTTTTTCTGGTGTTAGGAGCCCTTTTGTTCCTTTTCACATGTTATATAGTGGTTAAGGCAGGG

**C3H** 697 CTGAAAATTCTGGATGAAATTCAGGGCAGTCTATCAGAAGTAAAGCGGGGAGAGAGAGTAGGAGCAAGGAGAAACGGTAAGTATACAGGCCTTTCCAAGG

**129** 701 CTGAAAATTCTGGATGAAATTCAGGGCAGTCTATCAGAAGTAAAGCGGGGAGAGAGAGTAGGAGCAAGGAGAAACGGTAAGTATACAGGCCTTTCCAAGG

**C3H** 797 GTCTTGAACCCGAGGAAAAGTTAAGGTTAGGTAGGAATACCTGGAGAGAGATTAGAAGAAAAAGAGGAAAAAGGGAAAAGAAAAAAGATCGATTAGCGGA

**129** 801 GTCTTGAACCCGAGGAAAAGTTAAGGTTAGGTAGGAATACCTGGAGAGAGATTAGAAGAAAAAGAGGAAAAAGGGAAAAGAAAAAAGATCGATTAGCGGA

**C3H** 897 GGTCTCTAGGAGATACTCGTCACTAGATGAGCTCAGGAAGCCAGCTCTTAGTAGCTCTGAAGCAAGTGAAGAATCCTCCTCTGAGGAAACAGACTGGGAG

**129** 901 GGTCTCTAGGAGATACTCGTCACTAGATGAGCTCAGGAAGCCAGCTCTTAGTAGCTCTGAAGCAAGTGAAGAATCCTCCTCTGAGGAAACAGACTGGGAG

**C3H** 997 GAAGAAGCAGCCCATTACCAGCCAGCTAATTGGTCAAGAAAAAAGCCAAAAGCGGCTGGCGAAAGTCAGCGTACTGTTCAACCTCCCGGCAGTCGGTTTC

**129** 1001 GAAGAAGCAGCCCATTACCAGCCAGCTAATTGGTCAAGAAAAAAGCCAAAAGCGGCTGGCGAAAGTCAGCGTACTGTTCAACCTCCCGGCAGTCGGTTTC

**C3H** 1097 AAGGTCCGCCCTATGCGGAGCCCCCGCCCTGCGTAGTGCGTCAGCAATGCGCAGAGAGGCAATGCGCAGAGAGGTGCGCAGAGAGGCAGTGCGCAGACAG

**129** 1101 AAGGTCCGCCCTATGCGGAGCCCCCGCCCTGCGTAGTGCGTCAGCAATGCGCAGAGAGGCAATGCGCAGAGAGGTGCGCAGAGAGGCAGTGCGCAGACAG

**C3H** 1197 GTGCGCAGAGAGGCAGTGCGCAGAGAGGCAGTGCGCAGACTCATTCATTCCCCGAGAGGAACAAAAGAAAATAGAACAGGCATTTCCAGTCTTTGAAGGA

**129** 1201 GTGCGCAGAGAGGCAGTGCGCAGAGAGGCAGTGCGCAGACTCATTCATTCCCCGAGAGGAACAAAAGAAAATAGAACAGGCATTTCCAGTCTTTGAAGGA

**C3H** 1297 GCCGAGGGTGGGCGTGTCCACGCTCCGGTAGAATACGTACAGATTAAGGAAATTGCCGAGTCGGTTC**GTAAATACGGAACCAATGCTAATTTCACCTTGG**

**129** 1301 GCCGAGGGTGGGCGTGTCCACGCTCCGGTAGAATACGTACAGATTAAGGAAATTGCCGAGTCGGTTCGTAAATACGGAACCAATGCTAATTTCACCTTGG

**C3H** 1397 **TGCAGTTAGACAGGCTCGCTGGTATGGCACTAACGCCTGCTGATTGGCAGACGGTTGTAAAAGCCGCTCTTCCTAGTATGGGCAAATATATGGAATGGAA**

**129** 1401 TGCAGTTAGACAGGCTCGCTGGTATGGCACTAACGCCTGCTGATTGGCAGACGGTTGTAAAAGCCGCTCTTCCTAGTATGGGCAAATATATGGAATGGAA

**C3H** 1497 **AGC**GCTTTGGCACGAAGCTGCACAGGCGCAGGCCCGAGCAAACGCAGCTGCTTTGACTCCAGAGCAGATAGATTGGACTTTTGACTTGTTAACGGGTCAG

**129** 1501 AGCGCTTTGGCACGAAGCTGCACAGGCGCAGGCCCGAGCAAACGCAGCTGCTTTGACTCCAGAGCAGAGAGATTGGACTTTTGACTTGTTAACGGGTCAG

**C3H** 1597 GGGGCTTATTCTGCTGATCAGACAAGCTACCATTGGGGAGCTTATGCCCAGATTTCTTCCACGGCTATTAGGGCCTGGAAGGCGCTCTCTCGAGCAGGTG

**129** 1601 GGAGCTTATTCTGCTGATCAGACAAACTACCATTGGGGAGCTTATGCCCAGATTTCTTCCACGGCTATTAGGGCCTGGAAGGCGCTCTCTCGAGCAGGTG

**C3H** 1697 AAACCACTGGTCAGTTAACAAAAATAATCCAGGGACCTCAGGAATCTTTCTCAGATTTTGTGGCCAGAATGACAGAGGCAGCAGAGCGTATTTTTGGAGA

**129** 1701 AAACCACTGGTCAGTTAACAAAAATAATCCAGGGACCTCAGGAATCTTTCTCAGATTTTGTGGCCAGAATGACAGAGGCAGCAGAGCGTATTTTTGGAGA

**C3H** 1797 GTCAGAGCAAGCTGCGCCTCTCATAGAACAGCTAATCTACGAGCAAGCCACAAAGGAGTGCCGAGCGGCCATAGCCCCAAGAAAGAACAAAGGCTTACAA

**129** 1801 GTCAGAGCAAGCTGCGCCTCTCATAGAACAGCTAATCTACGAGCAAGCCACAAAGGAGTGCCGAGCGGCCATAGCCCCAAGAAAGAACAAAGGCTTACAA

**C3H** 1897 GACTGGCTCAGGGTCTGTCGAGAGCTTGGGGGACCCCTTAGCAATGCAGGTTTAGCGGCTGCCATCCTTCAATCCCAAAACCGCTCCATGGGCAGAAATG

**129** 1901 GACTGGCTCAGGGTCTGTCGAGAGCTTGGGGGACCCCTTAGCAATGCAGGTTTAGCGGCTGCCATCCTTCAATCCCAAAACCGCTCCATGGGCAGAAATG

**C3H** 1997 ATCAGAGGACATGTTTTAACTGCGGAAAGCCTGGGCATTTTAAGAAAGATTGCAGAGCTCCAGATAAACAGGGAGGGGCTCTCACTCTTTGCTCTAAGTG

**129** 2001 ATCAGAGGACATGTTTTAACTGCGGAAAGCCTGGGCATTTTAAGAAAGATTGCAGAGCTCCAGATAAACAGGGAGGGACTCTCACTCTTTGCTCTAAGTG

**C3H** 2097 TGGCAAGGGTTATCATAGAGCTGACCAGTGTCGCTCTGTGAGGGATATAAAGGGCAGAATTCTTCCCCCACCTGATAGTCAATCAGCTGATGTGCCAAAA

**129** 2101 TGGCAAGGGTTATCATAGAGCTGACCAGTGTCGCTCTGTGAGGGATATAAAGGGCAGAATTCTTCCCCCACCTGATAGTCAATCAGCTGATGTGCCAAAA

**C3H** 2197 AACGGGTCACCGGGCCCTCGGTCCCAGGGCCCTCAAAGATATGGGAACCGGTTTGTCAGGACCCAGGAAGCAGTCAGAGAGACGACCCAGGAAGACCCAC

**129** 2201 AACGGGTCACCGGGCCCTCGGTCCCAGGGCCCTCAAAGATATGGGAACCGGTTTGTCAGGACCCAGGAAGCAGTCAGAGAGACGACCCAGGAAGACCCAC

**C3H** 2297 AAGGGTGGACCTGCGTGCCGCCTCCGACTTCCTATTAATGCCTCAAATGAGTATTCAGCCGGTGCCGGTGGAGCCTATACCATCCTTGCCCCCGGGAACC

**129** 2301 AAGGGTGGACCTGCGTGCCGCCTCCGACTTCCTATTAATGCCTCAAATGAGTATTCAGCCGGTGCCGGTGGAGCCTATACCATCCTTGCCCCCGGGAACC

**C3H** 2397 ATGGGCCTTATTCTCGGCCGAGGTTCACTCACCTTGCAGGGCTTAGTAGTCCACCCTGGAATTATGGATTGTCAACATTCCCCTGAAATACAGGTCCTGT

**129** 2401 ATGGGCCTTATTCTCGGCCGAGGTTCACTCACCTTGCAGGGCTTAGTAGTCCACCCTGGAATTATGGATTGTCAACATTCCCCTGAAATACAGGTCCTGT

**C3H** 2497 GCTCAAGCCCTAAAGGCGTTTTTTCTATTAGTAAAGGAGATAGGATAGCTCAGCTGCTGCTCCTCCCTGATAATACCAGGGAGAAATCTGCAGGACCTGA

**129** 2501 GCTCAAGCCCTAAAGGCGTTTTTTCTATTAGTAAAGGAGATAGGATAGCTCAGCTGCTGCTCCTCCCTGATAATACCAGGGAGAAATCTGCAGGACCTGA

**C3H** 2597 GATAAAGAAAATGGGCTCCTCAGGAAATGATTCTGCCTATTTGGTTGTATCTTTAAATGATAGACCTAAGCTCCGCCTTAAGATTAATGGAAAAGAGTTT

**129** 2601 GATAAAGAAAATGGGCTCCTCAGGAAATGATTCTGCCTATTTGGTTGTATCTTTAAATGATAGACCTAAGCTCCGCCTTAAGATTAATGGAAAAGAGTTT

**C3H** 2697 GAAGGCATCCTTGATACCGGAGCAGATAAAAGTATAATTTCTACACATTGGTGGCCCAAAGCATGGCCCACCACAGAGTCATCTCATTCATTACAGGGCC

**129** 2701 GAAGGCATCCTTGATACCGGAGCAGATAAAAGTATAATTTCTACACATTGGTGGCCCAAAGCATGGCCCACCACAGAGTCATCTCATTCATTACAGGGCC

**C3H** 2797 TAGGATATCAATCATGTCCCACTATAAGCTCCGTTGCCTTGACGTGGGAATCCTCTGAAGGGCAGCAAGGGAAATTCATACCTTATGTGCTCCCACTCCC

**129** 2801 TAGGATATCAATCATGTCCCACTATAAGCTCCGTTGCCTTGACGTGGGAATCCTCTGAAGGGCAGCAAGGGAAATTCATACCTTATGTGCTCCCACTCCC

**C3H** 2897 GGTTAACCTCTGGGGAAGGGATATTATGCAGCATTTGGGCCTTATTTTGTCCAATGAAAACGCCCCATCAGGAGGGTATTCAGCTAAAGCAAAAAATATC

**129** 2901 GGTTAACCTCTGGGGAAGGGATATTATGCAGCATTTGGGCCTTATTTTGTCCAATGAAAACGCCCCATCAGGAGGGTATTCAGCTAAAGCAAAAAATATC

**C3H** 2997 ATGGCAAAGATGGGTTATAAAGAAGGAAAAGGGTTAGGACATCAAGAACAGGGAAGGATAGAGCCCATCTCACCTAATGGAAACCAAGACAGACAGGGTC

**129** 3001 ATGGCAAAGATGGGTTATAAAGAAGGAAAAGGGTTAGGACATCAAGAACAGGGAAGGATAGAGCCCATCTCACCTAATGGAAACCAAGACAGACAGGGTC

**C3H** 3097 TGGGTTTTCCATAGCGGCCATTGGGGCAGCACGACCCATACCATGGAAAACAGGGGACCCAGTGTGGGTTCCTCAATGGCACCCATCCTCTGAAAAACTA

**129** 3101 TGGGTTTTCCATAGCGGCCATTGGGGCAGCACGACCCATACCATGGAAAACAGGGGACCCAGTGTGGGTTCCTCAATGGCACCTATCCTCTGAAAAACTA

**C3H** 3197 GAAGCTGTGATTCAACTGGTAGAGGAACAATTAAAATTAGGCCATATTGAACCCTCTACCTCACCTTGGAATACTCCAATTTTTGTAATTAAGAAAAAGT

**129** 3201 GAAGCTGTGATTCAACTGGTAGAGGAACAATTAAAATTAGGCCATATTGAACCCTCTACCTCACCTTGGAATACTCCAATTTTTGTAATTAAGAAAAAGT

**C3H** 3297 CAGGAAAGTGGAGACTGCTCCATGACCTCAGAGCCATTAATGAGCAAATGAACTTATTTGGCCCAGTACAGAGGGGTCTCCCTGTACTTTCCGCCTTACC

**129** 3301 CAGGAAAGTGGAGACTGCTCCATGACCTCAGAGCCATTAATGAGCAAATGAACTTATTTGGCCCAGTACAGAGGGGTCTCCCTGTACTTTCCGCCTTACC

**C3H** 3397 ACGTGGCTGGAATTTAATCATTATAGATATTAAAGATTGTTTCTTTTCTATACCTTTGTGTCCAAGGGATAGGCCCAGATTTGCCTTTACCATCCCCTCT

**129** 3401 ACGTGGCTGGAATTTAATCATTATAGATATTAAAGATTGTTTCTTTTCTATACCTTTGTGTCCAAGGGATAGGCCCAGATTTGCCTTTACCATCCCCTCT

**C3H** 3497 ATTAATCACATGGAACCTGATAAGAGGTATCAATGGAAGGTCTTACCACAGGGAATGTCCAATAGTCCTACTATGTGTCAACTTTATGTACAAGAAGCTC

**129** 3501 ATTAATCACATGGAACCTGATAAGAGGTATCAATGGAAGGTCTTACCACAGGGAATGTCCAATAGTCCTACTATGTGTCAACTTTATGTACAAGAAGCTC

**C3H** 3597 TTTTGCCAGTGAGGGAACAATTCCCCTCTTTAATTTTGCTCCTTTACATGGATGACATCCTCCTGTGCCATAAAGACCTTACCATGCTACAAAAGGCATA

**129** 3601 TTTTGCCAGTGAGGGAACAATTCCCCTCTTTAATTTTGCTCCTTTACATGGATGACATCCTCCTGTGCCATAAAGACCTTACCATGCTACAAAAGGCATA

**C3H** 3697 TCCTTTTCTACTTAAAACTTTAAGTCAGTGGGGTCTACAGATAGCCACAGAAAAGGTCCAAATTTCTGATACAGGACAATTCTTGGGCTCTGTGGTGTCC

**129** 3701 TCCTTTTCTACTTAAAACTTTAAGTCAGTGGGGTCTACAGATAGCCACAGAAAAGGTCCAAATTTCTGATACAGGACAATTCTTGGGCTCTGTGGTGTCC

**C3H** 3797 CCAGATAAGATTGTGCCCCAAAAGGTAGAGATAAGAAGAGATCACCTCCATACCTTAAATGATTTTCAAAAGCTGTTGGGAGATATTAATTGGCTCAGAC

**129** 3801 CCAGATAAGATTGTGCCCCAAAAGGTAGAGATAAGAAGAGATCACCTCCATACCTTAAATGATTTTCAAAAGCTGTTGGGAGATATTAATTGGCTCAGAC

**C3H** 3897 CCTTTTTAAAGATTCCTTCTGCTGAATTAAGGCCTTTGTTTAGTATTTTAGAAGGAGATCCTCATATCTCCTCCCCTAGGACTCTTACTCTAGCTGCTAA

**129** 3901 CCTTTTTAAAGATTCCTTCTGCTGAATTAAGGCCTTTGTTTAGTATTTTAGAAGGAGATCCTCATATCTCCTCCCCTAGGACTCTTACTCTAGCTGCTAA

**C3H** 3997 CCAGGCCTTACAAAAAGTGGAAAAAGCCTTACAGAATGCACAATTACAACGTATTGAGGATTCGCAGCCTTTCAGTTTGTGTGTCTTTAAGACAGCACAA

**129** 4001 CCAGGCCTTACAAAAAGTGGAAAAAGCCTTACAGAATGCACAATTACAACGTATTGAGGATTCGCAGCCTTTCAGTTTGTGTGTCTTTAAGACAGCACAA

**C3H** 4097 TTGCCAACTGCAGTTTTGTGGCAAAATGGGCCATTGTTGTGGATCCATCCAAACGTATCCCCAGCTAAAATAATAGATTGGTATCCTGATGCAATTGCAC

**129** 4101 TTGCCAACTGCAGTTTTGTGGCAAAATGGGCCATTGTTGTGGATCCATCCAAACGTATCCCCAGCTAAAATAATAGATTGGTATCCTGATGCAATTGCAC

**C3H** 4197 AGCTTGCCCTTAAAGGCCTAAAAGCAGCAATCACCCACTTTGGGCAAAGTCCATATCTTTTAATTGTACCTTATACCGCTGCACAGGTTCAAACCTTGGC

**129** 4201 AGCTTGCCCTTAAAGGCCTAAAAGCAGCAATCACCCACTTTGGGCAAAGTCCATATCTTTTAATTGTACCTTATACCGCTGCACAGGTTCAAACCTTGGC

**C3H** 4297 AGCCGCATCTAATGATTGGGCAGTTTTAGTTACCTCCTTTTCAGGAAAAGTAGATAACCATTATCCAAAGCATCCAATCTTACAGTTTGCCCAAAATCAA

**129** 4301 AGCCGCATCTAATGATTGGGCAGTTTTAGTTACCTCCTTTTCAGGAAAAATAGATAACCATTATCCAAAGCATCCAATCTTACAGTTTGCCCAAAATCAA

**C3H** 4397 TCTGTTGTGTTTCCACAAATAACAGTAAGAAACCCACTTAAAAATGGGATTGTGGTATATACTGATGGATCAAAAACTGGCATAGGTGCCTATGTGGCTA

**129** 4401 TCTGTTGTGTTTCCACAAATAACAGTAAGAAACCCACTTAAAAATGGGATTGTGGTATATACTGATGGATCAAAAACTGGCATAGGTGCCTATGTGGCTA

**C3H** 4497 ATGGTAAAGTGGTATCCAAACAATATAATGAAAATTCACCTCAAGTGGTAGAATGTTTAGTGGTCTTAGAAGTTTTAAAAACCTTTTTAGAACCCCTTAA

**129** 4501 ATGGTAAAGTGGTATCCAAACAATATAATGAAAATTCACCTCAAGTGGTAGAATGTTTAGTGGTCTTAGAAGTTTTAAAAACCTTTTTAGAACCCCTTAA

**C3H** 4597 TATTGTGTCAGATTCCTGTTATGTGGTAAATGCAGTAAATCTTTTAGAAGTGGCCGGAGTGATTAAGCCTTCCAGTAGAGTTGCCAATATTTTTCAGCAG

**129** 4601 TATTGTGTCAGATTCCTGTTATGTGGTAAATGCAGTAAATCTTTTAGAAGTGGCTGGAGTGATTAAGCCTTCCAGTAGAGTTGCCAATATTTTTCAGCAG

**C3H** 4697 ATACAATTAGTTTTGTTATCTAGAAGATCTCCTGTTTATATTACTCATGTTAGAGCCCATTCAGGCCTACCTGGCCCCATGGCTCTGGGAAATGATTTGG

**129** 4701 ATACAATTAGTTTTGTTATCTAGAAGATCTCCTGTTTATATTACTCATGTTAGAGCCCATTCAGGCCTACCTGGCCCCATGGCTCTGGGAAATGATTTGG

**C3H** 4797 CAGATAAGGCCACTAAAGTGGTGGCTGCTGCCCTATCATCCCCGGTAGAGGCTGCAAGAAATTTTCATAATAATTTTCATGTGACGGCTGAAACATTACG

**129** 4801 CAGATAAGGCCACTAAAGTGGTGGCTGCTGCCCTATCATCCCCGGTAGAGGCTGCAAGAAATTTTCATAATAATTTTCATGTGACGGCTGAAACATTACG

**C3H** 4897 CAGTCGTTTCTCCTTGACAAGAAAAGAAGCCCGTGACATTGTTACTCAATGTCAAAGCTGCTGTGAGTTCTTGCCAGTTCCTCATGTGGGAATTAACCCA

**129** 4901 CAGTCGTTTCTCCTTGACAAGAAAAGAAGCCCGTGACATTGTTACTCAATGTCAAAGCTGCTGTGAGTTCTTGCCAGTTCCTCATGTGGGAATTAACCCA

**C3H** 4997 CGCGGTATTCGACCTCTACAGGTCTGGCAAATGGATGTTACACATGTTTCTTCCTTTGGAAAACTTCAATATCTCCATGTGTCCATTGACACATGTTCTG

**129** 5001 CGCGGTATTCGACCTCTACAGGTCTGGCAAATGGATGTTACACATGTTTCTTCCTTTGGAAAACTTCAATATCTCCATGTGTCCATTGACACATGTTCTG

**C3H** 5097 GCATCATGTTTGCTTCTCCGTTAACTGGAGAAAAAGCCTCACATGTGATTCAACATTGTCTTGAGGCATGGAGTGCTTGGGGGAAACCCAGACTCCTTAA

**129** 5101 GCATCATGTTTGCTTCTCCGTTAACTGGAGAAAAAGCCTCACATGTGATTCAACATTGTCTTGAGGCATGGAGTGCTTGGGGGAAACCCAGACTCCTTAA

**C3H** 5197 GACTGATAATGGACCAGCTTATACGTCCCAAAAATTTCAGCAGTTCTGCCGTCAGATGGACGTAACCCACCTGACTGGACTTCCATACAACCCTCAAGGA

**129** 5201 GACTGATAATGGACCAGCTTATACGTCCCAAAAATTTCAGCAGTTCTGCCGTCAGATGGACGTAACCCACCTGACTGGACTTCCATACAACCCTCAAGGA

**C3H** 5297 CAGGGTATTGTTGAGCGTGCGCATCGCACCCTCAAAGCCTATCTTATAAAACAGAAGAGGGGAACTTTTGAGGAGACTGTACCCCGAGCACCAAGAGTGT

**129** 5301 CAGGGTATTGTTGAGCGTGCGCATCGCACCCTCAAAGCCTATCTTATAAAACAGAAGAGGGGAACTTTTGAGGAGACTGTACCCCGAGCACCAAGAGTGT

**C3H** 5397 CGGTGTCTTTGGCACTCTTTACACTCAATTTTTTAAATATTGATGCTCATGGCCATACTGCGGCTGAACGTCATTGTTCAGAGCCAGATAGGCCCAATGA

**129** 5401 CGGTGTCTTTGGCACTCTTTACACTCAATTTTTTAAATATTGATGCTCATGGCCATACTGCGGCTGAACGTCATTGTTCAGAGCCAGATAGGCCCAATGA

**C3H** 5497 GATGGTTAAATGGAAAAATGTCCTTGATAATAAATGGTATGGCCCGGATCCTATCTTGATAAGATCCAGGGGAGCTATCTGTGTTTTCCCACAGAATGAA

**129** 5501 GATGGTTAAATGGAAAAATGTCCTTGATAATAAATGGTATGGCCCGGATCCTATCTTGATAAGATCCAGGGGAGCTATCTGTGTTTTCCCACAGAATGAA

**C3H** 5597 GACAACCCATTTTGGGTACCAGAAAGACTCACCCGAAAAATCCAGACTGACCAAGGGAATACTAATGTCCCTCGTCTTGGTGATGTCCAGGGCGTCAATA

**129** 5601 GACAACCCATTTTGGGTACCAGAAAGACTCACCCGAAAAATCCAGACTGACCAAGGGAATACTAATGTCCCTCGTCTTGGTGATGTCCAGGGCGTCAATA

**C3H** 5697 ATAAAGAGAGAGCAGCGTTGGGGGATAATGTCGACATTTCCACTCCCAATGACGGTGATGTATAATGCTCAAGTATTCTCCTGCTTTTTTACCACTAACT

**129** 5701 ATAAAGAGAGAGCAGCGTTGGGGGATAATGTCGACATTTCCACTCCCAATGACGGTGATGTATAATGCTCAAGTATTCTCCTGCTTTTTTACCACTAACT

**C3H** 5797 GGGAACTGGGTTTGGCCTTAATTCAGACAGCCTTGGTTCTGTCTGGACAGGTCCAGATGACTGACACCATTAACACTTTGTCAGCCTCAGTGACTACAGT

**129** 5801 GGGAACTGGGTTTGGCCTTAATTCAGACAGCCTTGGTTCTGTCTGGACAGGTCCAGATGACTGACACCATTAACACTTTGTCAGCCTCAGTGACTACAGT

**C3H** 5897 CATAGATAAACAGGCCTCAGCTAATGTCAAGATACAGAGAGGTCTCATGCTGGTTAATCAACTCATAGATCTTGTCCAGATACAACTAGATGTATTATGA

**129** 5901 CATAGATAAACAGGCCTCAGCTAATGTCAAGATACAGAGAGGTCTCATGCTGGTTAATCAACTCATAGATCTTGTCCAGATACAACTAGATGTATTATGA

**C3H** 5997 CAAATAACTCAGCAGGGATGTGAACAAAAGTTTCCGGGATTGTGTGTTATTTCCATTCAGTATGTTAAATTTACTAGGGCAGCTAATTTGTCAAAAAGTC

**129** 6001 CAAATAACTCAGCAGGGATGTGAACAAAAGTTTCCGGGATTGTGTGTTATTTCCATTCAGTATGTTAAATTTACTAGGGCAGCTAATTTGTCAAAAAGTC

**C3H** 6097 TTTTTCAGTATATGTTACAGATCCTTCGAGAATTGAGACTTCAGGTCAACTCCACGCGCTTGGACCTGTCGCTGACCAAAGGATTACCCAATTGGATCTC

**129** 6101 TTTTTCAGTATATGTTACAGATCCTTCGAGAATTGAGACTTCAGGTCAACTCCACGCGCTTGGACCTGTCGCTGACCAAAGGATTACCCAATTGGATCTC

**C3H** 6197 CTCAGCATTTTCTTTCTTTAAAAAATTGGGTGGGATTAATATTATTTGGAGATACACGTTGCTGTGGATTAGTGTTGCTTCTTTGATTGGTCTGTAAGCT

**129** 6201 CTCAGCATTTTCTTTCTTTAAAAAATTGGGTGGGATTAATATTATTTGGAGATACACGTTGCTGTGGATTAGTGTTGCTTCTTTGATTGGTCTGTAAGCT

**C3H** 6297 TAAGGCCCAAACTAAGAGAGACAAGGTGGTTATTGCCCAGGCGCTTGCAGGACTAGAACATGGAGCTTCCCCTGATATATCTATGCTTAAGCAATAGGTC

**129** 6301 TAAGGCCCAAACTAAGAGAGACAAGGTGGTTATTGCCCAGGCGCTTGCAGGACTAGAACATGGAGCTTCCCCTGATATATCTATGCTTAAGCAATAGGTC

**C3H** 6397 GCTGGCCACTCAGCTCTTATATCTCACGAGGCTAGTCTCATTGCACGAGGTAGAGTGAGTGTGCTTCAGCAGCCCGAGAGAGTTGCAAGGCTAAGCACTG

**129** 6401 GCTGGCCACTCAGCTCTTATATCTCACGAGGCTAGTCTCATTGCACGAGATAGAGTGAGTGTGCTTCAGCAGCCCGAGAGAGTTGCAAGGCTAAGCACTG

**C3H** 6497 CAGTAGAAGGGCTCTGCGGCACATATGAGCCTATTCTAGGGAGACATGTCATCTTTCATGAAGGTTCAGTGTCCTAGTTCCCTTCCCCCAGGCAAAACGA

**129** 6501 CAGTAGAAGGGCTCTGCGGCACATATGAGCCTATTCTAGGGAGACATGT---------------------------------------------------

**C3H** 6597 CACGGGAGCAGGTCAGGGTTGCTCTGGGTAAAAGCCTGTAAGCCTAAGAGCTAATCCTGTACATGGCTCCTTTACCTACACACTGGGGATTTGACCTCTA

**129** 6549 ---------AGGTCAGGGTTGCTCTGGGTAAAAGCCTGTAAGCCTAAGAGCTAATCCTGTACATGGCTCCTTTACCTACACACTGGGGATTTGACCTCTA

**C3H** 6697 TCTCCACTCTCATTAATATGGGTGGCCTATTTGCTCTTATTAAAAGAAAAAGGGGGAACTGTGGGAAGCCGCCCCCACATTCGCCGACACAAGATGGCGC

**129** 6641 TCTCCACTCTCATTAATATGGGTGGCCTATTTGCTCTTATTAAAAGAAAAAGGGGGAACTGTGGGAAGCCGCCCCCACATTCGCCGTCACAAGATGGCGC

**C3H** 6797 TGACATCCTGTGTTCTAAGTTGGTAAACAAATAATCTGCGCATGAGCCAAGGGTATTTACGACTACTTGTACTCTGTTTTTCCCGTGAACGTCAGCTCGG

**129** 6741 TGACATCCTGTGTTCTAAGTTGGTAAACAAATAATCTGCGCATGAGCCAAGGGTATTTACGACTACTTGTACTCTGTTTTTCCCGTGAACGTCAGCTCGG

**C3H** 6897 CCATGGGCTGCAGCCAATCAGGGAGTGATGCGCCCTAGGCAATGGTTGTTCTCTTTAAAATAGAAGGGGTTTCGTTTTTCTCGCTCTCTTGCTTCCCTCT

**129** 6841 CCATGGGCTGCAGCCAATCAGGGAGTGATGCGCCCTAGGCAATGGTTGTTCTCTTTAAAATAGAAGGGGTTTCGTTTTTCTCGCTCTCTTGCTTCCCTCT

**C3H** 6997 CTTGCTTCTTACACTCTGGCCCGATAAAGATATAAGCAATAAAGCTTTGCCGTAGAAGATTCTGGTTGTTGTGTTCTTCCTGGCCGGTCGTGAGAACGCG

**129** 6941 CTTGCTTCTTACACTCTGGCCCGATAAAGATATAAGCAATAAAGCTTTGCCGTAGAAGATTCTGGTTGTTGTGTTCTTCCTGGCCGGTCGTGAGAACGCG

**C3H** 7097 TCGAATAACA

**129** 7041 TCGAATAACA
